# Editorial: Sepsis awareness month 2024

**DOI:** 10.3389/fmed.2026.1990102

**Published:** 2026-10-01

**Authors:** Bojan Jovanovic, Adi Hadzibegovic

**Affiliations:** Faculty of Medicine, University of Belgrade, Belgrade, Serbia

**Keywords:** antibio tics, biomarkers, hemodynamic management, precision medicine, sepsis

Sepsis remains one of the most formidable challenges in contemporary medicine. Despite substantial advances in critical care, antimicrobial therapy, organ support, and our understanding of host–pathogen interactions, sepsis continues to impose a major burden of mortality, long-term disability, and healthcare utilization worldwide. Its clinical heterogeneity, rapid progression, and complex immunometabolic biology make both early recognition and individualized treatment particularly difficult. Importantly, sepsis is not solely an intensive care problem: its impact extends from population-level disease surveillance and prevention to emergency diagnosis, source control, precision medicine, hemodynamic management, and post-acute recovery.

The Research Topic “Sepsis Awareness Month 2024” brings together eight contributions that reflect this breadth. Collectively, these studies emphasize a transition from viewing sepsis as a single clinical syndrome toward understanding it as a dynamic and heterogeneous process that requires better epidemiological characterization, earlier recognition, personalized risk assessment, timely treatment, and increasingly individualized therapeutic strategies.

One important dimension of this transition is improved characterization of the host immune response. Li et al. investigated longitudinal peripheral blood lymphocyte trajectories rather than relying on isolated measurements. Four distinct lymphocyte phenotypes were identified and externally validated, with the high-declining trajectory associated with greater disease severity and mortality. Their findings illustrate how dynamic immunological monitoring may provide information that static laboratory measurements cannot and may ultimately help identify patients who could benefit from immune-targeted therapies (Li et al.). Such an approach is particularly relevant as the concept of sepsis-associated immunosuppression becomes increasingly integrated into clinical thinking.

The drive toward precision medicine is further developed by Su et al., who applied artificial intelligence across heterogeneous patient cohorts to identify a minimal set of highly informative molecular biomarkers. Their work highlights CKAP4, FCAR, RNF4 and other population-specific markers and demonstrates the potential of computational approaches to reduce the enormous number of proposed sepsis biomarkers to clinically manageable signatures (Su et al.). The challenge ahead is to determine whether such highly accurate research models retain their performance prospectively and whether they can be translated into rapid and accessible bedside diagnostics.

While precision diagnostics continue to evolve, several fundamental aspects of sepsis management remain highly time dependent. Gretland et al. revisited the critical question of antimicrobial timing. Among 42 studies analyzed, 34 demonstrated an association between delayed antibiotic administration and increased mortality. At the same time, their review highlights the heterogeneity of sepsis and cautions against interpreting a universal 1-h target without consideration of clinical context (Gretland et al.). The central message is therefore not simply “antibiotics as rapidly as possible,” but rather rapid recognition combined with prompt, appropriate antimicrobial therapy while maintaining diagnostic accuracy and antimicrobial stewardship.

Appropriate resuscitation is similarly dependent on timing, dose, and individual physiology. In critically ill surgical patients with intra-abdominal sepsis, Popović et al. demonstrated that greater cumulative fluid balance during the first week of ICU treatment was associated with increased in-hospital mortality. Higher sodium and chloride concentrations were also associated with adverse outcomes, even when mean values remained largely within reference ranges (Popović et al.). These findings reinforce the evolving concept that intravenous fluids should be treated as drugs—with an indication, dose, duration, and potential toxicity—and that the resuscitation phase must eventually transition toward fluid restriction and de-resuscitation when clinically appropriate.

Individualization of cardiovascular support is explored from another perspective by Woodford and Marshall, who describe the use of a novel “pressure field” method to visualize the interaction between cardiac output and vascular tone in a patient with occult septic shock and severe myocardial dysfunction. The patient recovered despite requiring unusually high vasopressor and inotrope doses (Woodford and Marshall). Although evidence from case-based experience cannot establish efficacy, the report raises an important broader question: whether future shock management should move beyond population-derived pressure targets toward continuously individualized hemodynamic goals.

Source identification and control remain equally fundamental. Zhang et al. describe an unusual case in which an iatrogenic hematoma compressed the ureters, resulting in obstruction and urogenic sepsis. Recognition of the mechanical cause and emergency ureteral stenting resulted in marked clinical improvement (Zhang et al.). The case serves as a reminder that successful sepsis treatment requires more than physiological stabilization. Persistent or deteriorating sepsis should prompt repeated consideration of an uncontrolled or initially unrecognized infectious source.

Beyond established treatment paradigms, this Research Topic also considers potential future therapies. Dimic et al. review the role of glucagon-like peptide-1 receptor agonists (GLP-1RAs) in sepsis, highlighting possible anti-inflammatory, antioxidant, metabolic, mitochondrial, endothelial, and muscle-protective effects (Dimic et al.). These pleiotropic mechanisms are scientifically compelling, particularly given the central role of metabolic and endothelial dysfunction in sepsis. Nevertheless, translation from experimental observations to clinical therapy will require carefully designed prospective trials before GLP-1RAs can be regarded as a therapeutic component of sepsis care.

Finally, progress in sepsis cannot depend solely on interventions at the bedside. Gatica and Kissoon demonstrate how official health records and data-science methods can quantify sepsis incidence and mortality at subnational levels in Chile (Gatica and Kissoon). Their local-to-global strategy highlights geographic differences that may be hidden within national estimates and provides a framework for allocating public health resources where the burden is greatest. This is particularly important because effective sepsis programs require reliable surveillance, regional benchmarking, and locally adapted healthcare policies.

Taken together, the articles in this Research Topic illustrate that improving sepsis outcomes will require simultaneous progress at several levels: population surveillance, rapid recognition, dynamic immune and molecular phenotyping, timely antimicrobial treatment and source control, careful fluid administration, individualized hemodynamic support, and development of novel therapies. They also demonstrate a central tension in modern sepsis care: the need to act rapidly while recognizing biological and clinical heterogeneity.

Sepsis awareness should therefore extend beyond recognition of the syndrome itself. Awareness must increasingly mean recognition of its heterogeneity, its changing physiology over time, and the need to match interventions to the right patient at the right moment. The studies collected in “Sepsis Awareness Month 2024” contribute to this transition from standardized sepsis management toward more data-informed, dynamic, and personalized care.

